# The pharmacokinetics of tigecycline in critically ill adult patients undergoing CVVHD

**DOI:** 10.1186/2197-425X-3-S1-A403

**Published:** 2015-10-01

**Authors:** E Rypulak, J Sysiak, M Borys, P Piwowarczyk, B Potrec, M Czuczwar

**Affiliations:** Department of Anesthesiology and Intensive Therapy, Lublin Medical University, Lublin, Poland

## Introduction

There is growing evidence that pharmakokinetic parameters of commonly used antibiotics may not be optimal in the critically ill patients. Significant changes of pharmacokinetic parameters, including volume of distribution and clearance, could be expected. Recently, dose increases of antibiotics in the ICU have been advocated to maximise their antibacterial activity, especially when severe infections with high bacterial load and/or multidrug resistance are suspected.

## Objectives

The objective of this study was to describe the pharmacokinetics of tigecycline in critically ill patients receiving continuous venovenous haemodialysis (CVVHD) and to evaluate the frequency of pharmacokinetic/pharmacodynamic target attainment with high dosing strategy (200 mg loading dose and 100 mg/12h).

## Methods

This was a prospective observational study in critically ill patients receiving CVVHD and administered tigecycline in high doses. Serial tigecycline concentrations in plasma were measured 2, 4, 8 and 12 hours during antibiotic therapy. Tigecycline pharmacokinetic parameters were calculated using a non-compartmental approach.

## Results

Over the study period, 16 patients treated with CVVHD received tigecycline at a high dose. There were no patients requiring tigecycline discontinuation or dose reduction because of adverse events.

## Conclusions

Tigecycline was well tolerated at a higher than standard dose in a cohort of critically ill patients with severe infections requiring CVVHD.The pharmacokinetic-pharmacodynamic profile of dosing regimen tested in this study might be helpful in selecting the appropriate dose of tigecycline for patients receiving CVVHD.

## Grant Acknowledgment

This study was supported by Lublin Medical University (grant no. DS 152)

